# Job satisfaction and turnover intention among Iraqi doctors - a descriptive cross-sectional multicentre study

**DOI:** 10.1186/s12960-015-0014-6

**Published:** 2015-04-19

**Authors:** Saad Ahmed Ali Jadoo, Syed Mohamed Aljunid, Ilker Dastan, Ruqiya Subhi Tawfeeq, Mustafa Ali Mustafa, Kurubaran Ganasegeran, Sami Abdo Radman AlDubai

**Affiliations:** United Nations University-International Institute of Global Health (UNU-IIGH), International Centre for Case-Mix and Clinical Coding (ITCC), National University of Malaysia Medical Centre (UKMMC), Jalan Yaacob Latiff, 56000 Cheras Kuala Lumpur, Malaysia; International Centre for Case-Mix and Clinical Coding (ITCC), National University of Malaysia Medical Centre (UKMMC), Jalan Yaacob Latiff, 56000 Cheras Kuala Lumpur, Malaysia; Department of Economics, Izmir University of Economics, Izmir, Turkey; Department of Public Health, Faculty of Medicine, Tiqrit University, Tiqrit, Iraq; Medical Department, Tengku Ampuan Rahimah Hospital (HTAR), Jalan Langat, Klang, Selangor Malaysia; Department of Community Medicine, International Medical University (IMU), Bukit Jalil, Kuala Lumpur, Malaysia

**Keywords:** Iraqi doctors, Health human resources migration, Turnover intention, Job satisfaction

## Abstract

**Background:**

During the last two decades, the Iraqi human resources for health was exposed to an unprecedented turnover of trained and experienced medical professionals. This study aimed to explore prominent factors affecting turnover intentions among Iraqi doctors.

**Methods:**

A descriptive cross-sectional multicentre study was carried out among 576 doctors across 20 hospitals in Iraq using multistage sampling technique. Participants completed a self-administered questionnaire, which included socio-demographic information, work characteristics, the 10-item Warr-Cook-Wall job satisfaction scale, and one question on turnover intention. Descriptive and bivariate and multiple logistic regression analyses were conducted to identify significant factors affecting turnover intentions.

**Results:**

More than one half of Iraqi doctors (55.2%) were actively seeking alternative employment. Factors associated with turnover intentions among doctors were low job satisfaction score (odds ratio (OR) = 0.97; 95% confidence interval (CI): 0.95, 0.99), aged 40 years old or less (OR = 2.9; 95% CI: 1.74, 4.75), being male (OR = 4.2; 95% CI: 2.54, 7.03), being single (OR = 5.0; 95% CI: 2.61, 9.75), being threatened (OR = 3.5; 95% CI: 1.80, 6.69), internally displaced (OR = 3.1; 95% CI: 1.43, 6.57), having a perception of unsafe medical practice (OR = 4.1; 95% CI: 1.86, 9.21), working more than 40 h per week, (OR = 2.3; 95% CI: 1.27, 4.03), disagreement with the way manager handles staff (OR = 2.2; 95% CI: 1.19, 4.03), being non-specialist, (OR = 3.9, 95% CI: 2.08, 7.13), and being employed in the government sector only (OR = 2.0; 95% CI: 1.09, 3.82).

**Conclusion:**

The high-turnover intention among Iraqi doctors is significantly associated with working and security conditions. An urgent and effective strategy is required to prevent doctors’ exodus.

## Background

The global healthcare sector has been facing major challenges through retention problems and labor shortages [1]. Retention of healthcare workers is a significant concern, because it is extremely costly due to the hiring and training of new workers [2,3]. Besides, labor shortage is detrimental to healthcare system performance and services [4]. According to the World Health Organization (WHO) [5], healthcare labor shortages are common globally; however, this phenomenon is crucial in countries where healthcare performance indicators are the worst. WHO in its report during the Third Global Forum on Human Resources for Health indicated that by 2035 the world will be short of 12.9 million healthcare workers [5].

Causes of labor shortage have been documented in literature. The main causes include prolonged conflicts, political and social instabilities, inadequate investment in healthcare sector, and migration of healthcare workers [6,7]. The civil and regional wars jointly with security disturbances in countries such as Lebanon, Somalia, Liberia, Iran, Afghanistan, and other similar nations had resulted in a mass exodus and death of the healthcare personnel accompanied with serious health and economic consequences for the population [8-13].

Job dissatisfaction has been stated to be one of the most major and consistent predictor of turnover intention and migration of healthcare workers [1,14-17]. Job satisfaction of healthcare workers arises from relations between experience and work environment and is greatly essential for healthcare workers’ motivation [18], retention, and performance [19]. Existing studies confirmed a relationship between the low job satisfaction, the low motivation, and the turnover intention and a relationship between the low job satisfaction and the actual physician migration [20-23]. The concept of turnover intention was broadly studied in items of “conceptualizations, forms, antecedents, consequences, intermediate linkages, mediators, moderators, as well as applications” [24]. However, turnover intention is seldom precisely defined in most of the prominent research outputs [25]. This practice was justified by Bothma [25] to the assumption that people had probably perceived the term to be self-explanatory. Turnover intention was conceptualized by Cotton and Turtle [26] as “an individual’s perceived probability of staying or leaving an employing organization”. Also, Hom and Griffeth [27] defined turnover intention as“ the relative strength of an individual’s intent toward voluntary permanent withdrawal from an organization”. Tett and Meyer [28] “argued that turnover intention can be used as a valid proxy for actual labor turnover”. Several authors viewed turnover intention as “the final step in the decision-making process before a person actually leaves a workplace, in which members actively consider quitting and searching for alternative jobs or professions [28,29]”.

Most of the instruments available to measure the turnover intention had used only a limited number of scale items such as the measure based on the Mobley, Horner, and Hollingsworth theory [30]; the scale items were: (1) I think a lot about leaving the organization, (2) I am actively searching for an alternative to the organization, and (3) As soon as it is possible, I will leave the organization. Some other measures are lacking information on the metric properties as in the case of the turnover intention scale (TIS-6) [25]. Therefore, the common trend among various researchers was to use single-item scales [31-33].

The fact that turnover intention was shown to be the strongest predictor of actual leaving or actual turnover among healthcare personnel was documented [34]. Oluwafemi [24] indicated that “the early detection of employee’s job dissatisfaction through turnover intention measure would be greatly helpful to resolve the problem of exodus before it exacerbates”. Therefore, turnover intention for the purpose of this study implies the Iraqi physicians’ intention to potentially quit their present jobs to pursue other alternative employment.

### Overview of the Iraqi doctors’ situation

Iraq is an oil-exporting country in the Middle East. It had reached the middle-income status in the 1970s [35,36]. In the period from 1980 to 1988, healthcare financing was reduced greatly due to the Iran–Iraq War [37]. Throughout the 1990s, a strict ban imposed on the Iraqi regime after the invasion of Kuwait affected the health system in Iraq negatively. The economic collapse, feelings of persecution, job stress, abuses of human rights, and the isolation from the world’s scientific environment were the main predictors to leave abroad among Iraqi doctors [38]. Nevertheless, after the US-led invasion of Iraq in 2003, the already weakened health system was exposed to further damage due to organized and widespread acts of looting and destruction [39,40], “accompanied with a systematic targeting of the brightest, most distinguished and most highly regarded doctors and scientists” [41]. Up to date, all available sources about the actual number of physicians who have been kidnapped, assassinated, migrated, and even those who are still working in Iraq are estimated figures [42,43].

Many references [44-48] estimated that around 20,000 Iraqi physicians had fled out since the 2003 invasion. The World Health Organization reported that Iraq is continuing to face a severe shortage of healthcare professionals; it is ranked 95 globally and is in the bottom of the list of the regional countries [49]. To compensate for the continuing brain-drain process in Iraq, the Ministry of Health and Ministry of Higher Education have taken an immediate action after the 2003 invasion to add more medical schools. The number of medical schools rose from just 7 before the 2003 invasion to 12 in 2004 and 20 in 2007 and peaked up to 23 by 2012 [42]. UNESCO’s report of 2004 indicated that the total number of medical students in the 12 medical schools was 18 018; this means roughly 2,000 to 3,000 had graduated every year [42,50]. Consequently, the physician-to-population ratio showed a slow and sometimes fluctuated growth over the last decade. The World Health Organization, Ministry of Health, and World Bank estimated the ratio of 5.5 per 10,000 populations in 1990, 5.0 in 2002, 6.3 in 2004, 6.6 in 2005, 6.1 in 2006, and 6.9 in 2012 [49,51,52]. However, the national rate of physician per population in 2012 is much lower than the global (14.2 per 10,000) and regional rate (10.9 per 10,000) and far fewer than neighbouring countries such as Iran (8.9), Kuwait (17.9), Turkey (15.4), Syria (15.0), Saudi Arabia (9.4), and Jordan (24.5) [49]. A special report presented by Webster [48] in 2013 referred to about 24, 745 physicians currently in practice (according to government figures); Iraq’s physician-to-population ratio is about 60% lower than the average for the other 23 nations in WHO’s Eastern Mediterranean Region. Burnham et al. [8] in their findings of 12 tertiary care centres study in Iraq, “they considered the 61% of specialists who left their hospital posts a major loss of human capital from Iraq’s hospital sector, a loss that is likely to require some years to fully replace”.

The political events and the embargo imposed on Iraq in the 1990s led to a scarcity of research about Iraqi doctors. However, little has been done after the invasion of Iraq in 2003. Al-Khalisi [48] reported that 50% of doctors surveyed via emails in Iraq showed interest to leave in the near future. The same author reported that 60% of those who are already abroad had left the country due to security issues. The security breakdown, destruction of infrastructures, workload, bad management, corruption, politicization of the healthcare system, and the lack of strategic planning in all fields were the most notable features in the last decade [39,40,53].

Since June 2014, an escalating rate of violence in central and northern Iraq made medical staff and healthcare facilities a target of repeated and unwarranted military actions [54-56]. It seems that under the full inability of the government over the last decade to save the human resources, the turnover intention among Iraqi doctors became inevitable. Accordingly, our study is very important, relevant, and timely to examine the perceived turnover intention of Iraqi doctors to leave their work or their country and the factors associated with turnover intention, particularly those related to job satisfaction. Thus, by examining predictors of turnover intentions, policy makers in Iraq and in countries with similar conditions may be able to take steps to better retain their healthcare workforce.

## Methods

A cross-sectional study was conducted in Iraq from 1 January until 30 June 2014. A representative sample of doctors was selected by assuming that the turnover intention is 50% among Iraqi doctors [24] using the following formula:

$$ N=\left[Z{\alpha}^2\times P\times Q\;/\;{(M.E.)}^2\right] $$

So, *n* = (1.96)^2^ × (0.50) × (0.50)/(0.04)^2^ = 600. Non-response correction = 10%. Thus, the total sample size was 660. A multistage sampling technique was recruited to collect the data: first, we divided Iraq into five geographical regions (north, central, west, south, and the capital city). Then, we selected one province randomly from each geographical region. Then, two districts from each selected province were selected randomly. Then, from each selected district, two hospitals were selected. Twenty hospitals have been included: five teaching hospitals, five tertiary hospitals, five general hospitals, and five district hospitals. A list of the doctors was obtained from each hospital. An average of 33 doctors per hospital was selected randomly from each hospital, and they were contacted personally by the researcher team. Each eligible respondent received a copy of the questionnaire manually. The contact number of the data collector and his email were provided to each respondent in case they needed any clarification. All the physicians of Iraqi nationality who completed their medical bachelor degree in Iraq, who were employed in the selected hospitals during the study period, and who were willing to participate were included. The chief medical officer (CMO), deputy CMO, hospital manager, and deputy hospital manager were excluded. The total number of doctors who answered the self-administered questionnaire was 576(response rate = 87.3%).

### Study instrument

The self-administered questionnaire used in this study included questions on the socio-demographic information, work characteristics, a 10-item Warr-Cook-Wall (WCW) job satisfaction questionnaire with seven-point Likert scales [57], and one question on turnover intention [30]. All questions were in English because English is the language of teaching and instruction in all Iraqi medical schools and hospitals [58]. The questionnaire was test piloted among 20 doctors.

### Dependent variables

Physicians’ turnover intention was assessed by asking the doctors to score one statement “*I’m actively seeking alternative employment*” adopted from Mobley, Horner, and Hollingsworth [30]. Responses were measured with a four-point Likert-type scale ranging from 1 = “strongly disagree” to 4 = “strongly agree” and dichotomized into (0) disagreed (original categories 1, 2) and (1) agreed (original categories 3, 4).

### Independent variables

Job satisfaction was measured with a 10-item job satisfaction scale, which employed a seven-point Likert-type scale ranging from 1 = “very dissatisfied” to 7 = “very satisfied”. Cronbach’s alpha in our sample was excellent (0.97). We used the total sum score of the 10 items (in range of 10 to 70) to obtain the overall job satisfaction measure. Our predictors included also the socio-demographic factors and work characteristics. Some other predictors were inspired from Iraq’s situation which may affect the physician’s turnover intention.

The responses were given as either (1) “Yes” or (0) “No” in response to the following questions: “Do you have children?”, “Did you lose a family member in Iraq due to violence?”, and “Have you been threatened in Iraq to violence?” The “internally displaced” doctor was defined as the doctor who has forced to move from his usual place of residency and work in another place inside the home country, because of armed conflict, generalized violence, and human rights violations [59]. The internal displacement was assessed by asking participants, “Have you been displaced internally?” Responses were given as (1) “yes” and (0) “no”.

Additionally, the four-point Likert-type scale ranging from 1 = “strongly disagree” to 4 = “strongly agree” and dichotomized into (0) disagreed (original categories 1, 2) and (1) agreed (original categories 3, 4) was recruited to assess *the relationship between doctors and their patients* by asking the doctors to score the statement, “The doctor-patient-relationship is excellent”; *the safety and security of medical practice in Iraq* by asking doctors to score the statement, “Current medical practice in Iraq is safe”; *the relationship between doctors and their senior manager* was assessed by asking the doctors to score the statement, “The way the senior manager handles the staff is effective”; and *the training and educational opportunities* was assessed by asking the doctors to score the statement, “The training and educational opportunities in your interest area were good”.

Furthermore, *the working hours per week* were defined and categorized as (1) “40 hours per week” and (0) “more than 40 hours per week”. *Number of years spent at their work or the same facility* was categorized as (0) less than 10 years and (1) being 10 years and more. *The current professional level* was defined as whether the doctor has completed at least one of the postgraduate degrees (diploma, master, doctorate) or has only the bachelor of medicine and surgery and categorized as (1) specialist and (0) non-specialist. *Type of employment* was defined and categorized as (1) when the doctor was employed in “the government only” and (0) “mixed” when the doctor was employed in the government and also worked in the private sector.

### Ethical considerations

Our study protocol was approved by the Izmir Economic University’s Ethics Committee in 2014, Code number (B.30.2.IEU.0.05.05-020-014) and by respective authorities of the selected hospitals where data collection took place. Confidentiality was assured and written consent was obtained from all respondents.

### Data analysis

Data collected were analysed using Statistical Package for Social Science (SPSS) program version 20.0 (SPSS Inc., Chicago IL, USA) [60]. Normality tests were done, and all the quantitative data were found to be normally distributed. Frequency distribution and descriptive statistics of socio-demographic variables and work characteristics were obtained to provide the sample profile. Furthermore, a descriptive analysis was performed concerning the overall job satisfaction and the 10 items of job satisfaction to obtain the means and standard deviation (SD). An independent-sample *t*-test was run to determine if there were differences in overall job satisfaction between doctors who were actively seeking alternative employment (turnover intentions) and those who were not. Chi square tests were used in the bivariate analysis for binary or categorical variables. Significant factors predicting turnover intention on bivariate analysis (*p* value <0.05) were included in the multivariate model. Multiple logistic regression analysis (Enter technique) was performed to identify significant predictors of turnover intentions. In “Enter technique”, the variables in the models which are not significant are removed one by one until a satisfactory model is obtained. Odds ratio and 95% confidence interval were calculated. An alpha level of *p* < 0.05 is considered to be statistically significant.

## Results

### Descriptive and general characteristics of related factors

Five hundred and seventy-six completed questionnaires were analysed. Mean age (±SD) was 40.43 years (±8.59), and the age ranged from 27 to 56 years old. Two thirds of doctors were married (64.2%), and 53.8% were females (and 51.2% had children). Out of the total sample, 26.6% reported they had lost one or more of their close relatives in Iraq due to violence, 54.3% of them have been threatened, and 39.1% have been internally displaced at least once because of violence. Two thirds (66.8%) of the doctors were from the urban region, and the majority (59.5%) had spent more than 10 years at their work or the same facility. Out of the 576 doctors, 255 (44.3%) were employed in the government sector only, 39.8% were specialists, and 46.4% worked more than 40 h per week. Four hundred and seven (70.7%) of the doctors reported that their relation with the patients was excellent, and 385 (66.8%) of them considered their medical practice in Iraq was unsafe or risky. Forty percent disagreed with the way their senior manager handled staff, and 45.1% were dissatisfied with their training and educational opportunities (Table 1).

**Table 1:** **Respondents’ socio demographic and work characteristics (** ***n*** **= 576)**

**Respondent’s characteristics**	**Category**	***N***	**%**
Age	≤40 years old	285	49.5
	>40 years old	291	50.5
Gender	Male	266	46.2
	Female	310	53.8
Marital status	Married	370	64.2
	Single	206	35.8
Presence of children	Yes	295	51.2
	No	281	48.8
Residency	Rural	211	36.6
	Urban	365	63.4
Loss of family member	Yes	153	26.6
	No	423	73.4
Exposure to threat	Yes	313	54.3
	No	263	45.7
Internally displaced doctors	Yes	225	39.1
	No	351	60.9
Medical practice in Iraq is safe	Agree	191	33.2
	Disagree	385	66.8
The current professional level	Specialist	229	39.8
	Non-specialist	347	60.2
The way managers handle staff was effective	Agree	343	59.5
	Disagree	233	40.5
Doctor-patient relationship is excellent	Agree	169	29.3
	Disagree	407	70.7
Duration of work	< or = 10 years	154	26.7
	>10 years	422	73.3
Training and educational opportunities	Satisfied	316	54.9
	Dissatisfied	260	45.1
Hours of work/week	>40 h	267	46.4
	40 h	309	53.6
Type of employment	Government only	255	44.3
	Government and private	321	55.7

### Job satisfaction and turnover intention

The mean (SD) value on the total job satisfaction score was 42.44 (SD = 14.87). The level of satisfaction on “the freedom to choose own method of working” was the highest followed by opportunities to use their abilities, remuneration, working hours, and variation in work. The lowest satisfaction score was reported for physical working conditions, recognition for good work, overall job satisfaction, amount of responsibility, and the cooperation with colleagues and fellow workers (Table 2).

**Table 2:** **Descriptive statistics of the 10 items and overall job satisfaction scale**

**No.**	**Job satisfaction statements (WCW)**	**Mean**	**SD**	**Min.**	**Max.**
1	Physical working conditions	3.81	1.63	1	7
2	Freedom to choose your own method of working	4.60	1.79	1	7
3	Your colleagues and fellow workers	4.26	1.61	1	7
4	Recognition you get for good work	3.89	1.62	1	7
5	Amount of responsibility you are given	4.22	1.72	1	7
6	Your remuneration, i.e. income	4.40	1.70	1	7
7	Opportunity to use your abilities	4.54	1.69	1	7
8	Your hours of work	4.30	1.75	1	7
9	Amount of variety in your job	4.30	1.72	1	7
10	Taking everything into consideration, how do you feel about your job?	4.12	1.79	1	7
*11*	*Overall scale job satisfaction*	*42.44*	*14.87*	*10*	*70*

WCW, Warr-Cook-Wall job satisfaction scale.

An independent-sample *t*-test was run to determine if there were differences in overall job satisfaction between doctors who were actively seeking alternative employment (turnover intention) and their counterparts. There were no outliers in the data, as assessed by inspection of a boxplot. Overall job satisfaction scores for each level of turnover intention were normally distributed, as assessed by the Kolmogorov–Smirnov test (*p* > 0.05). Overall job satisfaction was more among doctors who were not actively seeking alternative employment (*m* = 49.38, SD = 12.52) than doctors who were actively seeking alternative employment (*m* = 36.80, SD = 14.25), a statistically significant difference (*m* = 12.58, 95% CI (10.39, 14.77), *t* (570.385) = 11.269, *p* < 0.001). More than one half of the doctors (318, 55.2%) agreed that they were actively seeking alternative employment compared to (250, 44.8%) who disagreed.

### Factors associated with turnover intention in bivariate analysis

Cross tabulation indicated that only doctors who were aged 40 years old or less, (chi square test (*χ*^2^) = 63.79, *p* < 0.001), being male *(χ*^2^ = 41.10, *p* < 0.001), being single (unmarried, divorced, and widowed) (*χ*^2^ = 26.18, *p* < 0.001), being threatened (*χ*^2^ = 11.54, *p* =0.001), internally displaced (*χ*^2^ = 31.69, *p* < 0.001), perceived work unsafe or risky, (*χ*^2^ = 4.91, *p* = 0.027), and being non-specialist (*χ*^2^ = 80.57, *p* < 0.001), disagreed with the way the manager handles the staff (*χ*^2^ = 71.02, *p* < 0.001), employed in the government sector only (*χ*^2^ = 50.72, *p* < 0.001), and working more than 40 h per week (*χ*^2^ = 61.29, *p* < 0.001) were significantly associated with the turnover intention (Table 3).

**Table 3:** **Bivariate analysis of predictors in turnover intention**

**Factors**	**Category**	**Yes**	**No**	***χ*** ^**2**^	***p***
		**318(55.2%)**	**250(44.8%)**		
Age	≤40 years	205(71.9)	80(28.1)	63.79	*<0.001*
	>40 years	113(38.8)	178(61.2)		
Gender	Male	185(69.5)	81(30.5)	41.10	*<0.001*
	Female	133(42.9)	177(57.1)		
Marital status	Single	143(69.4)	63(30.6)	26.18	*<0.001*
	Married	175(47.3)	195(52.5)		
Presence of children	Yes	161(54.6)	134(45.4)	0.10	0.755
	No	157(55.9)	124(44.1)		
Residency	Rural	117(55.5)	94(44.5)	0.008	0.929
	Urban	201(55.1)	164(44.9)		
Loss of family member	Yes	91(59.5)	62(40.5)	1.54	0.215
	No	227(53.7)	196(46.3)		
Being threatened	Yes	193(55.2)	120(44.8)	11.54	*0.001*
	No	125(47.5)	138(52.5)		
Displaced internally	Yes	157(69.8)	68(30.2)	31.69	*<0.001*
	No	161(45.9)	190(54.1)		
Medical practice is safe	Disagree	225(58.4)	160(41.6)	4.91	*0.027*
	Agree	93(48.7)	98(51.3)		
Hours of work/week	>40 Hours	194(72.7)	73(27.3)	61.30	*<0.001*
	≤40 Hours	124(40.1)	185(59.9)		
Way manager handles staff	Disagree	178(76.4)	55(23.6)	71.02	*<0.001*
	Agree	140(40.8)	203(59.2)		
Doctor-patient relationship	Agree	90(53.3)	79(46.7)	0.37	0.543
	Disagree	228(56.0)	179(44.0)		
The professional level	Non specialist	244(70.3)	103(29.7)	80.57	*<0.001*
	Specialist	74(32.3)	155(67.7)		
Duration of work	≤10 Years	93(60.4)	61(39.6)	0.10	0.748
	>10 Years	225(53.3)	197(46.7)		
Training opportunities	Satisfied	170(53.8)	146(46.2)	0.56	0.453
	Dissatisfied	148(56.9)	112(43.1)		
Employment	Government only	183(71.8)	72(28.2)	50.72	*<0.001*
	Mixed	135(42.1)	186(57.9)		
Over all job satisfaction		Mean	SD		
	With intention	36.80	14.25		
	Without intention	49.38	12.52		

*p* < 0.05 is statistically significant for data in italics.

### Factors associated with turnover intention in multiple logistic regression

Table 4 presents the final model of the multivariable logistic regressions. Overall job satisfaction (odds ratio (OR) = 0.97, 95% CI: 0.95 to 0.99) with the other 10 variables was associated significantly with “turnover intention” (*p* < 0.05). The doctors who disagreed with the way managers handle the staff (OR = 2.19, 95% CI: 1.19 to 4.03) and working more than 40 h per week (OR = 2.26, 95% CI: 1.27 to 4.03) had the lowest odds ratios. While the doctors who are single (OR = 5.05, 95% CI: 2.61 to 9.75) and being male (OR = 4.23, 95% CI: 2.54 to 7.03) had the highest odds ratios. The Hosmer and Lemeshow test indicated a good fit (*p* = 0.320). The total model was significant (*p* = 0.001) and accounted for 58.4% of variance (Nagelkerke *R* square = 0.584).

**Table 4:** **Factors associated with turnover intention in multiple logistic regression**

**Variables**	***B***	**SE**	**Wald**	***p*** **value**	**OR**	**95% CI**
Overall job satisfaction	−0.033	0.012	8.01	0.004	0.97	0.95–0.99
40 years old or less	1.056	0.256	16.97	0.000	2.87	1.74–4.75
More than 40 years old					Reference	
Male	1.441	0.259	30.90	0.000	4.23	2.54–7.03
Female					Reference	
Single	1.619	0.336	23.23	0.000	5.05	2.61–9.75
Married					Reference	
Being threatened	1.243	0.335	13.78	0.000	3.47	1.80–6.69
Not threatened					Reference	
Internally displaced	1.118	0.390	8.22	0.004	3.06	1.43–6.57
No					Reference	
Unsafe medical practice	1.420	0.408	12.09	0.001	4.12	1.86–9.21
Safe					Reference	
>40 h/week	0.822	0.296	7.71	0.005	2.28	1.27–4.06
< or = 40 h/week					Reference	
Disagreement with the way managers handle staff	0.785	0.310	6.39	0.011	2.19	1.19–4.03
Agree					Reference	
Non-specialist	1.349	0.314	18.50	0.000	3.85	2.08–7.13
Specialist					Reference	
Employed in government only	0.712	0.321	4.931	0.026	2.04	1.09–3.82
Mixed					Reference	
Constant	−3.719	0.811	21.01	0.000	0.024	

OR, odds ratio.

## Discussion

In the last decades, developing countries have seen healthcare professionals leaving the profession or migrating to developed countries [61]. This pattern is a particular problem in Iraq where the healthcare system has faced a catastrophic collapse since 1991. Iraqi doctors left the country due to falling wages during the sanction years, between 1990 and 2003, and because of the worsening security conditions after the 2003 invasion [62]. Even though the migration rates have slowed more recently, it was estimated that about half of the doctors have already left Iraq after the 2003 invasion, and most healthcare providers have significant turnover intentions or to leave the country [46]. It has been documented that the strongest predictor of an actual departure is developing an intention to leave the job [34]. An effective strategy for dealing with this crisis would be to identify health professionals’ turnover intention and take steps to reverse this intention. Due to the length of time need to train new physicians, it is important to retain existing ones in order to avoid serious public consequences [2,3,34].

In this study, more than half of the doctors surveyed (55%) were actively seeking alternative employment (turnover intention). Various factors related to this intention were examined. Findings revealed significant associations between turnover intention and job satisfaction, violence, and a number of demographic variables of age, gender, marital status, and a number of work-related variables of positional tenure, working hours, internal displacement, unsafe practice, managerial efficiency, and hospital type.

In this study, job satisfaction was negatively associated with turnover intention. The findings are similar to other studies done in Saudi Arabia [21], Lebanon [22], Ghana [33], Palestine [63], Pakistan [64], and China [65]. It is essential that policy makers and healthcare managers gain a better understanding of the causes of job dissatisfaction in the medical profession in order to develop effective retention strategies.

In this study, more than half of all healthcare professionals (54%) felt threatened, and 67% believed that the conditions of their medical practice were not sufficiently safe. The perception of being threatened and unsafe workplace due to violence are important predictors of turnover intention among the health professionals. Heponiemi et al. [66] found that the highest levels of turnover intentions were among physicians who had encountered a workplace physical violence. Additionally, reduced job satisfaction and a decline in the quality of job performance were reported among male junior physicians who were facing verbal and physical abuse from patients or their caretakers, respectively [67].

In Iraq, insecurity has been an important driver for external and internal migration of health professionals [8,48]. Burnham et al. [8] estimated that violent event rates associated with the migration of doctors have been increasing; migrations from Iraq were greatest in 2006, a time of remarkably intense violence, and the number of specialists at Baghdad hospitals declined by 24% between 2004 and 2007. To retain the existing healthcare workforce in Iraq, the results of this study clearly suggest that more attention should be paid to prevailing violence and increasing the security associated with the workplace [23].

In this study, age, gender, and marital status were associated with turnover intention among Iraqi doctors. Younger physicians were more likely to indicate a turnover intention compared to older doctors. This corresponds well with previous findings among physicians and other healthcare providers [13,21,22,66,65,68]. Older doctors may have stronger personal ties and may be more satisfied with their work and, therefore, less likely to contemplate leaving [13,21,68].

The literature is not consistent in terms of the relationship between gender and doctors’ turnover intention. However, this study showed that males had higher levels of turnover intention compared to females. This finding could be due to cultural issues such as the traditional gender roles, which attribute achievement and adventurousness of males. These opportunities are culturally less available for females with families who are usually less mobile and are less able to migrate or to quit their job [22,23,65,69]. Previous studies reported similar results among male physicians and nurses to have higher likelihood of turnover intentions compared to their female counterparts in China and Lebanon respectively [65,22].

Another significant predictor of turnover intention is marital status; unmarried doctors were more likely to indicate turnover intention. Similar findings were reported by Tai et al. [70] and El-Jardali et al. [22]. This finding could be explained by the fact that single healthcare professionals generally have fewer family responsibilities, thus making them more mobile. Moreover, work-related issues may be further affecting doctors’ turnover intention. The WHO reported that poor working conditions affect the level of satisfaction and retention [71]. Another finding of this study showed that doctors who are working more than 40 h per week were more likely to have turnover intention. Similarly, Heponiemi et al. [72] pointed out that physicians in Finnish “who had been on active call more than 40 hours per month had more turnover intentions than the other physicians”. Also Malik et al. [73] found that the doctors in Pakistan could be more satisfied with their jobs and have less intentions to leave their jobs when able to manage their work and life activities.

This study showed that internal displacement was positively associated with turnover intention. Around 40% of the participants were forced to move from their usual place of residency to work in far places within the country. Morton and Burnham [74] reported that such moves would be negatively related to job satisfaction and positively related to job stress and turnover intention. This study found that non-specialist doctors and those who considered their senior managers ineffective are more likely to have commitment issues or turnover intention than others. Similarly, Ahmad and Riaz [64] found a positive correlation between the attitudes of an immediate boss and the turnover intention of a subordinate. They argued that “the autocratic management style had negative relationship with intent to stay”. Koelewijn et al. [75] also indicated that physicians who perceive a high level of bureaucracy and lacking of influence on hospital policy are more likely to entertain dissatisfaction with management style. Furthermore, we found that physicians who are working in the public sector reported high-turnover intention compared to those having dual employment. Boerjan et al. [76] indicated that a double load created by working in a highly demanding job and being an apprentice in this profession at the same time may further exacerbate junior doctors’ intention to leave. Therefore, supporting new hires and non-specialist doctors in their healthcare roles may help decrease their turnover intention, particularly in public hospitals which are usually under-resourced and yet serve the poorer population in majority [77].

### Limitations of study

There are some limitations to this study. First, we analysed measured intentions to leave rather than actual turnovers, but actual behavioural measures of the Iraqi doctors may be different from intentional measures. Further, response bias is a possible bias, because we had no information about the non-respondents and if they differed in some criteria from the respondents. This study is also limited by the security situation in the country which affected the accessibility to the hospitals and districts. Many doctors preferred to complete the questionnaire in the privacy of their own home rather than at the hospital. Ethnic and sectarian conflict in Iraq peaked in 2006 and has had a major role in the migration and displacement of a large number of doctors; however, we omitted ethnic and religious affiliations from the questionnaire due to the sensitivity of these issues. Although we have test piloted the English version of the questionnaire, the language barrier could be a limitation because the native language of the respondent is Arabic. Finally, the study is a cross-sectional study which cannot establish a causal relationship between the variables.

### Policy implication

Healthcare workers’ turnover intention and migration are major problems facing the health industry, especially in countries suffering from prolonged war and civil conflicts coupled with political and economic decline like Iraq. We recommend providing effective regulations to protect healthcare staff from violent actions. The Iraqi government is now reviewing a policy of not issuing certificates to medical students for the duration of postgraduate training and practising to prevent their migration for few years. However, we believe that such a procedure should be accompanied with comprehensive reforms such as standardizing medical practices and medical procedure costs for the public and private sectors, improving working conditions, reducing the workload, offering competitive wages and benefit packages, and introducing new payment mechanisms such as pay for performance or casemix and diagnosis-related group systems. Moreover, the building up of non-monetary incentives is necessary to attract doctors by reducing the problems caused by work-related issues. We suggest that healthcare management should recognize and reward doctors who stay longer. Supervisor support programmes should be improved. It is recommended that healthcare managers should consider the current employee assistance programmes and to improve professional hospital administration. Administrators could provide career ladders, communication, and training programmes to make hospital and clinic environment more attractive, especially to the high-turnover-prone group of younger, non-specialist, and less tenured doctors. The above recommended actions may help to improve the retention of doctors working in public hospitals.

## Conclusion

It is concluded that intention to leave among Iraqi doctors was high, and it was significantly associated with job dissatisfaction and feeling unsecured, working conditions, and socio-demographic factor conditions. More than one half of the participants were actively seeking alternative employment. The results may be useful for policy makers and health administrators wishing to stop more doctors’ exodus or to retain the existing healthcare professionals in Iraq. To achieve this, urgent and concrete strategies must be developed focusing on the job security and job factors related to satisfaction. A particular attention should be given to the younger and single doctors. Improving working conditions, reducing work load, creating a safer working environment, and training senior managers to support their healthcare staff are very important actions. It is important to give particular attention to those who had high risk of turnover intention, e.g. young, single, and male, and those with less tenure, and those who worked at the public healthcare sector. This can be achieved by improving motivation and commitment through offering competitive wages, providing intrinsic rewards related to work schedule and conditions, and non-monetary support, such as training and supervision.

## Abbreviations

CMO: Chief medical officer
WCW: Warr-Cook-Wall
WHO: World Health Organization
TIS-6: Turnover intention scale
CI: Confidence interval
*χ*^2^: Chi square test
OR: Odds ratio
SD: Standard deviation
*n*: Number of all respondents
*B*: Unstandardized coefficients
SE: Standard error
*p* value: Level of significance
